# Integration of Two Diploid Potato Linkage Maps with the Potato Genome Sequence

**DOI:** 10.1371/journal.pone.0036347

**Published:** 2012-04-27

**Authors:** Kimberly J. Felcher, Joseph J. Coombs, Alicia N. Massa, Candice N. Hansey, John P. Hamilton, Richard E. Veilleux, C. Robin Buell, David S. Douches

**Affiliations:** 1 Department of Crop and Soil Sciences, Michigan State University, East Lansing, Michigan, United States of America; 2 Department of Plant Biology, Michigan State University, East Lansing, Michigan, United States of America; 3 Department of Horticulture, Virginia Tech, Blacksburg, Virginia, United States of America; Leuven University, Belgium

## Abstract

To facilitate genome-guided breeding in potato, we developed an 8303 Single Nucleotide Polymorphism (SNP) marker array using potato genome and transcriptome resources. To validate the Infinium 8303 Potato Array, we developed linkage maps from two diploid populations (DRH and D84) and compared these maps with the assembled potato genome sequence. Both populations used the doubled monoploid reference genotype DM1-3 516 R44 as the female parent but had different heterozygous diploid male parents (RH89-039-16 and 84SD22). Over 4,400 markers were mapped (1,960 in DRH and 2,454 in D84, 787 in common) resulting in map sizes of 965 (DRH) and 792 (D84) cM, covering 87% (DRH) and 88% (D84) of genome sequence length. Of the mapped markers, 33.5% were in candidate genes selected for the array, 4.5% were markers from existing genetic maps, and 61% were selected based on distribution across the genome. Markers with distorted segregation ratios occurred in blocks in both linkage maps, accounting for 4% (DRH) and 9% (D84) of mapped markers. Markers with distorted segregation ratios were unique to each population with blocks on chromosomes 9 and 12 in DRH and 3, 4, 6 and 8 in D84. Chromosome assignment of markers based on linkage mapping differed from sequence alignment with the Potato Genome Sequencing Consortium (PGSC) pseudomolecules for 1% of the mapped markers with some disconcordant markers attributable to paralogs. In total, 126 (DRH) and 226 (D84) mapped markers were not anchored to the pseudomolecules and provide new scaffold anchoring data to improve the potato genome assembly. The high degree of concordance between the linkage maps and the pseudomolecules demonstrates both the quality of the potato genome sequence and the functionality of the Infinium 8303 Potato Array. The broad genome coverage of the Infinium 8303 Potato Array compared to other marker sets will enable numerous downstream applications.

## Introduction

World-wide, potato is one of the most important food crops ranking third in total production behind wheat and rice [1]. However, cultivated potato (*Solanum tuberosum*) is an autotetraploid (2*n* = 4*x* = 48) which complicates both genetic/genomic studies as well as breeding efforts to improve important traits such as disease/pest resistance, processing quality and nutritional value. Multiple linkage maps have been constructed for potato in an effort to better understand the potato genome, develop markers for marker assisted breeding, and facilitate map-based cloning [2], [3], [4], [5]. Most potato linkage maps have been generated from diploid populations to simplify genetic segregation and to incorporate polymorphism from wild species and primitive cultivars. These maps range in size from 606 cM [2] to 1120 cM [4] and contain as few as 85 markers [6] and as many as 10,000 markers [5]. Potato linkage maps have been constructed from many types of markers, including isozymes, Restriction Fragment Length Polymorphisms (RFLPs), Simple Sequence Repeats (SSRs), Amplified Fragment Length Polymorphisms (AFLPs) and more recently, Single Nucleotide Polymorphisms (SNPs) [2], [7], [5], [8]. High frequencies of SNPs have been demonstrated in several crop species including maize [9], soybean [10] and rice [11] providing abundant variability to develop markers for marker assisted breeding.

In the last decade, the rapid evolution of next-generation sequencing technologies and associated bioinformatic pipelines has made it possible both technically and financially to generate genome sequences for many agronomically important crop species including rice (*Oryza sativa*) [12], maize (*Zea mays*) [13], soybean (*Glycine max*) [14] and potato (*Solanum tuberosum*) [15]. High density SNP maps have been developed for rice [16] and maize [17] which has increased our understanding of genome influence on crop performance. Thus, the publication of the potato genome sequence and the subsequent development and release of the Infinium 8303 Potato Array [18], [19] provide various opportunities to improve our understanding of the structure and function of the potato genome and to bridge the gap between genomics and applied breeding. We have genotyped two diploid potato populations using the Infinium 8303 Potato Array and created two linkage maps to validate the array. In addition, concordance between our linkage maps and the 12 pseudomolecules constructed by the Potato Genome Sequencing Consortium (PGSC) was tested in order to validate the potato genome sequence.

## Results and Discussion

### Fabrication of the Infinium 8303 Potato Array

SNPs for the Infinium array were selected from a set of biallelic, high confidence SNPs identified from transcriptome sequencing of six cultivated potato cultivars using either Sanger or Illumina transcriptome sequencing [18]. As we used SNPs derived from transcriptome not genome sequence data from only six cultivars, we can not assess allele frequency in SNP selection. Initial design of the array included 10,000 SNPs; however, due to allocation of two probes to detect transversion SNPs and assay failures, the final version of the “Infinium 8303 Potato Array” comprised 8,303 functional markers including 3,018 from candidate genes of interest, 536 from potato genetic markers and 4,749 selected for maximum genome coverage (**Table S1**). SNPs were distributed across the 12 chromosomes (**Figure S1**), providing abundant representation of the genes on all 12 chromosomes. Some SNPs (697) were on superscaffolds not anchored to the genome sequence and thus not present within the 12 pseudomolecules generated by the PGSC and used in this study. The pseudomolecules are representations of the 12 potato chromosomes generated by stitching superscaffolds, scaffolds and contigs into a contiguous sequence anchored to the chromosomes using both *in silico* and genetic mapping data as described previously [15]. SNPs (6,351 total) were distributed among 3,591 annotated genes, with a maximum of 29 SNPs occurring in a single gene (**Table S2**). Of the SNPs within annotated PGSC v3.4 genes [15], 5,538 SNPs were within the coding sequence (CDS) and 817 were within annotated untranslated regions (UTRs). The remainder of the SNPs (1,524) occurred in unannotated genes predicted from transcriptome sequencing and thus may represent genes missing within the PGSC v3.4 gene annotation dataset.

### Development of two diploid segregating populations

Two segregating, diploid potato populations (F_1_) derived from crosses with DM1-3 516 R44 (hereafter referred to as DM) were genotyped with the Infinium 8303 Potato Array. The female parent for both populations was DM, the doubled monoploid line used to generate the potato genome sequence [15]. DM is a homozygous diploid (2*n* = 2*x* = 24) derived from a heterozygous accession of *Solanum tuberosum* Group Phureja [20] using anther culture to generate a monoploid (2*n* = 1*x* = 12); leaf discs of the monoploid were subsequently placed in a tissue culture callus/regeneration protocol to induce spontaneous chromosome doubling resulting in a homozygous doubled monoploid. By using DM to generate a draft potato genome sequence, the PGSC was able to overcome the challenge of heterozygosity with respect to genome assembly. The use of DM as a parent in our diploid population also simplified the mapping process. Because DM is homozygous, all informative meioses occurred in the male parent. Therefore, a single linkage map was generated for each population representing the marker order in each male parent.

Population D84 (*n* = 92, from Michigan State University) resulted from a cross between DM and 84SD22. Breeding line 84SD22 (also called T704, [2], [21]) is a heterozygous diploid (*S. tuberosum*×*S. chacoense* hybrid, **Figure S2**) that was shown to have a higher percentage (59%) of polymorphic SNPs than other diploid clones crossed to DM in the Michigan State University potato breeding program (data not shown). Population DRH (*n* = 92, from Virginia Tech) was derived from a cross between DM and RH89-039-16 (hereafter referred to as RH). Breeding line RH is a heterozygous diploid with both *S. tuberosum* Groups Tuberosum and Phureja in its pedigree (see [15] supplemental data). The DRH population was selected for mapping because the RH clone has been used in genome sequencing [15] and because several genetic maps for RH exist [5], [8], [22], permitting comparisons to our SNP-based map of RH.

### Utility of the Infinium 8303 Potato Array

In general, the number of mapped markers per chromosome corresponded to chromosome size with the largest chromosome (1) represented by the greatest number of markers (**Table 1**). One obvious departure from this trend was chromosome 3 in DRH with only 88 mapped markers. The proportion of mapped markers within candidate genes, previously mapped genetic markers or at random genome locations, was similar in both populations and corresponded to the distribution of markers in the Infinium 8303 Potato Array (**Table 2**).

**Table 1:** Comparison of markers mapped in two diploid populations, DRH and D84.

	No. of markers						Length				
	All segregating markers^z^			Mapped markers			cM		Mb^y^		
Chrm.	DRH	D84	Common	DRH	D84	Common	DRH	D84	DRH	D84	DM^x^
1	268	279	114	121	76	14	125.0	98.2	80.8	81.3	81.5
2	208	270	103	97	55	17	78.9	53.3	45.5	46.0	47.1
3	88	239	26	64	46	6	78.1	61.2	47.8	47.7	47.9
4	230	186	74	105	53	12	88.7	90.6	63.6	63.6	64.3
5	144	158	52	55	46	9	99.9	65.0	46.9	46.8	47.0
6	213	216	110	90	59	19	65.8	65.3	52.4	54.7	55.0
7	146	245	52	66	49	5	69.6	46.8	53.2	53.2	53.4
8	147	183	57	74	48	11	71.1	66.8	43.2	43.1	43.6
9	164	195	62	89	57	8	99.9	68.9	52.9	52.5	53.6
10	115	131	51	66	43	14	81.8	63.3	52.0	51.8	52.3
11	131	171	45	74	50	8	75.7	47.5	41.5	41.9	42.3
12	106	181	41	43	55	4	30.8	65.2	54.1	58.9	59.1
Total	1960	2454	787	944	637	127	965.3	792.1	633.8	641.6	647.2

z Includes co-segregating markers plus mapped markers; 14 ungrouped markers and their 15 co-segregating markers from population DRH were not included in this list.

y Length spanned by the northern- and southern-most markers.

x Total length.

**Table 2:** Distribution of mapped markers in DRH, D84 and the Infinium 8303 Potato Array.

	% of markers	% of markers	% of markers
	in candidate	in previously	at selected
	genes	mapped markers	genomic locations
D84	33	4	62
DRH	34	5	60
Infinium 8303 Potato Array	36	6	57

After filtering to remove non-informative markers (**Table S3**), we observed 1,989 and 2,454 segregating markers in the DRH and D84 populations, respectively. Because the female parent in both populations (DM) was homozygous, all informative meioses occurred in the male parents (RH and 84SD22) and are equivalent in number to the number of segregating markers. However, pair-wise comparisons revealed 1,031 and 1,817 co-segregating loci in the DRH and D84 populations, respectively. After the removal of co-segregating loci and assignment of markers to linkage groups, the final number of unique mapped loci for each population was 944 (DRH) and 637 (D84) (**Table 1**). Including the co-segregating markers, over 4,400 markers were mapped including 787 markers common to both populations (**Table 1**). Map sizes were 965 cM (DRH) and 792 cM (D84) (**Table 1**). Previous map sizes for the RH clone ranged from 773 cM [5] to 857 cM [22] both smaller than the SNP-based map of RH generated in this study (**Table 3**). This was likely due to greater genome coverage with the SNP array as the Infinium 8303 Potato Array was designed to cover approximately 650 Mb of potato genome sequence. Although the potato genome sequence is 727 Mb, the DM pseudomolecules are smaller (647.2 Mb) (**Table 1**) as not all scaffolds could be anchored to a chromosome. Thus, the DRH map (633.8 Mb) covers 98% of the pseudomolecule length and 87% of the genome sequence length. Similarly, the D84 map (641.6 Mb) covers 99% of the pseudomolecule length and 88% of the genome sequence length. Van Os et al. [5] noted a strong clustering of markers in their map of the RH clone which resulted in uneven genome coverage. Furthermore, the 944 distinct marker loci comprising our DRH SNP map represented nearly double the number of unique loci (549 recombination bins) generated by van Os et al. [5] (**Table 3**). Thus, using the Infinium 8303 Potato Array, we were able to generate a larger map, with more unique loci and greater genome coverage than previous maps.

**Table 3:** Comparison of four diploid potato linkage maps: D84 and DRH maps (SNP based) and the ultra-high density (UHD) maps (AFLP-based [5]).

	No. of markers	No. of polymorphic	Co-segregating	Ungrouped	No. of unique	Map size
Map	screened	markers	markers^z^	markers	loci mapped	(cM)
D84	8303	2454	1817	0	637	792
DRH	8303	1989	1031	14	944	965
UHD (RH map)	10,000	3413	2772	92	549	773
UHD (SH map)	10,000	4187	3549	69	569	751

z The number of co-segregating markers in the UHD maps was calculated by subtracting the number of filled bins from the number of markers used for map construction.

**Figure 1:**
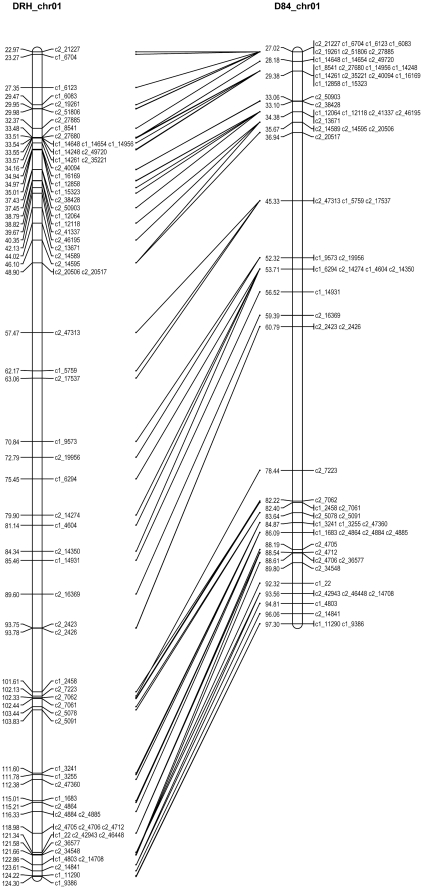
Alignment of chromosome 1 from DRH (left) and D84 (right).

The D84 linkage map was smaller than the DRH map despite the fact that it covered roughly the same proportion of the genome (**Table 1** **,** **Figure 1**). In a comparison of the two maps using the 787 markers that were common to both, we estimated the DRH linkage map to be 890 cM whereas the D84 linkage map was 641 cM (28% smaller than DRH) (**Table S4**). These data imply reduced recombination in D84. The male parent of this population was 84SD22, an *S. tuberosum*×*S. chacoense* hybrid that has a 30% reduction in recombination based on gene centromere mapping [21]. Bonierbale et al. [2] demonstrated reduced recombination in a different 84SD22-derived population. Gebhardt et al. [3] also noted significant reduction in map length with inter-specific compared to intra-specific crosses, which was attributed to reduced recombination. Therefore, the smaller linkage map we observed for the D84 population compared to the DRH population (which derived from cultigens within *S. tuberosum*) follows previous trends.

Distorted segregation ratios are common in potato mapping studies with a wide range in the reported percentage of mapped loci exhibiting aberrant segregation ratios: 25% [6], 28% [2] and 27 to 40% [3]. The percentage of mapped markers with distorted segregation was 4% and 9% in the DRH and D84 populations, respectively (**Table 4**). However, the same calculation performed with entire marker sets (i.e. mapped markers plus co-segregating markers) resulted in an increase in the percentage of markers with distorted segregation (DRH = 6% and D84 = 21%). This difference was due to co-segregating markers at locations where distorted segregation occurred and was more pronounced in the D84 population. Despite which markers were used in the analysis, the D84 population had a greater percentage of markers with distorted segregation. A similar phenomenon was observed between an *S. tuberosum*-derived population (27% of loci with distorted segregation) and an inter-specific population with *Solanum spegazzinii* in its pedigree (40% of loci with distorted segregation) [3]. In their study, Gebhardt et al. [3] suggested that inter-specific hybrids suffer not only from reduced recombination but also preferential selection for certain allelic combinations resulting in distorted segregation. As we cannot identify the origin (*S. tuberosum* or *S. chacoense*) of the alleles in the D84 population, we cannot determine if there is preferential selection of alleles based on species of origin. Hybrid breakdown (resulting in seedling death) has been observed among *S. chacoense* hybrids [23] and an *S. chacoense* mutant causing death at the cotyledon stage was also identified [24]; both are phenomena which could contribute to distorted segregation. In our study, any seedlings that did not survive and produce minitubers were not included in the mapping population. Therefore, we cannot rule out the contribution of hybrid breakdown or mutant alleles to the distorted segregation ratios that were observed.

**Table 4:** Markers with distorted segregation ratios in populations DRH and D84.

	All segregating markers^z^				Mapped markers^y^			
	No. of markers with		% of markers with		No. of markers with		% of markers with	
	distorted segregation		distorted segregation^x^		distorted segregation		distorted segregation^x^ ^z^	
Chrm.	DRH	D84	DRH	D84	DRH	D84	DRH	D84
1	1	1	0.4	0.4	1	1	0.8	1.3
2	0	1	0.0	0.4	0	1	0.0	1.8
3	0	160	0.0	66.9	0	9	0.0	19.6
4	0	122	0.0	65.6	0	17	0.0	32.1
5	1	0	0.7	0.0	1	0	1.8	0.0
6	1	123	0.5	56.9	1	15	1.1	25.4
7	0	1	0.0	0.4	0	1	0.0	2.0
8	0	115	0.0	62.8	0	14	0.0	29.2
9	27	0	16.5	0.0	11	0	12.4	0.0
10	0	0	0.0	0.0	0	0	0.0	0.0
11	0	0	0.0	0.0	0	0	0.0	0.0
12	83	0	78.3	0.0	24	0	55.8	0.0
Total	113	523	5.8	21.3	38	58	4.0	9.1

z Includes co-segregating markers plus mapped markers: 1960 (DRH) and 2454 (D84).

y Total number of mapped markers: 944 (DRH) and 637 (DRH).

x Percentage of markers per chromosome.

The markers with distorted segregation were unique to each population and were not localized to the same genomic regions for the two populations. The majority of the markers with distorted segregation were found in blocks on chromosomes 9 and 12 in DRH and on chromosomes 3, 4, 6 and 8 in D84 (**Table 4**). The size of these blocks ranged from 0 cM (blocks of co-segregating loci on chromosome 4 in D84) to 19.5 cM (**Table 5**). Individual markers with distorted segregation were located both at the ends of and within the chromosome maps. Bonierbale et al. [2] also found blocks of markers with distorted segregation on chromosomes 6 and 8 in an *S. chacoense*-derived population, consistent with what was observed in the D84 population.

**Table 5:** Position and size of blocks with distorted segregation.

		Position of blocks with	Size of block
Chromosome	Population	distorted segregation (cM)	(cM)
9	DRH	60.4–62.3	1.9
9	DRH	75.8–78.7	2.9
9	DRH	84.9–85.6	0.7
12	DRH	0.0–12.3	12.3
12	DRH	23.1–23.9	0.8
			
3	D84	0.0–7.5	7.5
4	D84	0.0–19.5	19.5
4	D84	20.7	0.0
4	D84	23.2	0.0
4	D84	25.7	0.0
6	D84	0.0–18.7	18.7
8	D84	0.0–14.5	14.5
8	D84	18.4–21.1	2.7

### Validation of the PGSC pseudomolecules

**Figure 2:**
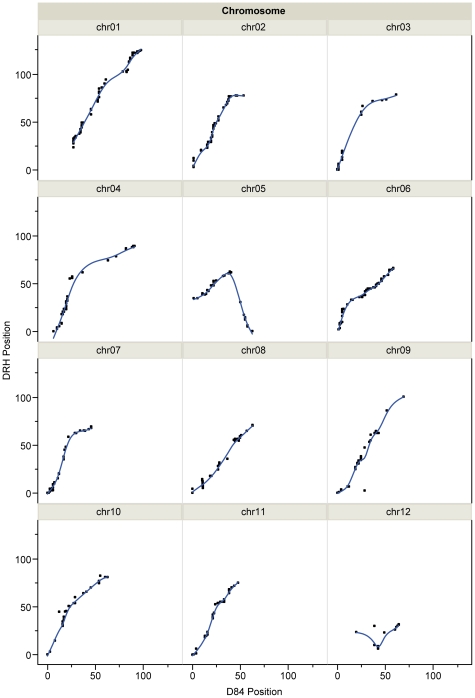
Graphs of DRH SNP marker position (cM) vs. D84 SNP marker position (cM) for all 12 potato chromosomes.

In order to compare the maps for both populations, we graphed the DRH marker positions against D84 marker positions for the 787 markers common to both populations. If marker order was identical in both populations, we would expect a straight line with a slope of 1. This is largely the case with the exceptions of chromosomes 5 and 12 (**Figure 2**). For chromosome 12, there were few data points. This may have contributed to the poor correlation and obscured the cause for the lack of concordance. By inspecting the cM position of common markers on chromosome 5, we identified a block of markers in D84 that was both mis-oriented and placed at the wrong end of the chromosome compared to DRH. Bonierbale et al [2] also detected an inversion on chromosome 5 in an *S. chacoense*-derived map relative to the tomato map. Although we initially assumed that the D84 map was incorrect, further inspection of the data showed that the D84 map order reflected the order and orientation of the current pseudomolecules. Thus, it appears that the genome sequence at this location differs between the RH clone, which is derived from cultivated potato, and the 84SD22 clone, which is derived from the wild species *S. chacoense*. Hu et al. [25] also noted chromosome rearrangements between *Arabidopsis thaliana* and *A. lyrata* despite greater than 80% sequence identity between them. One should, therefore, exercise caution when extrapolating sequence data between species.

When marker locations on the linkage maps were compared with their assigned position on the potato pseudomolecules, some discordances were noted (**Table 6** **and Table S5**). The chromosome assignment of 1.2% (DRH) and 1.8% (D84) of the markers differed between the linkage map and the pseudomolecule. If a discrepancy between the linkage map and the physical assemblies included more than one marker, we inspected the positions of these markers in the superscaffolds to determine whether the observed incongruity reflected a mis-assembly (i.e., incorrect order and/or orientation of superscaffolds) in the construction of the pseudomolecules. None of the disconcordant markers could be attributed to errors in the construction of the pseudomolecules. Disconcordant markers were also viewed in GenomeStudio to determine if the SNP assay was good (i.e., tight progeny clusters around the parental genotypes) or if there were multiple progeny clusters which could be indicative of a paralog. Of the 71 disconcordant markers, four (DRH) and five (D84) may be attributed to a paralog (**Table S5**). The presence of paralogs could also explain why some markers included in the linkage maps (7% in DRH and 10% in D84) could not be anchored to the pseudomolecules (**Table 7**). Among the disconcordant markers, two and 22 had distorted segregation ratios in DRH and D84, respectively, and the annotation was enriched for genes of unknown function (44%) (**Table S5**).

**Table 6:** Summary of disconcordant markers for each of the mapping populations.

				% of unique	% of disconcordant markers	
	No. of disconcordant markers^y^			disconcordant	per markers segregating^x^	
Chrm.^z^	DRH	D84	Total Unique	markers (59)	DRH	D84
1	2	2	4	6.8	0.7	0.7
2	1	0	1	1.7	0.5	0
3	1	3	4	6.8	1.1	1.3
4	1	3	2	3.4	0.4	1.6
5	3	1	2	3.4	2.1	0.6
6	2	5	5	8.5	0.5	2.3
7	5	6	9	15.2	2.7	2.4
8	1	1	2	3.4	0.7	0.5
9	2	5	7	11.9	1.2	2.6
10	7	9	12	20.3	6.1	6.9
11	1	1	2	3.4	0.8	0.6
12	0	9	9	15.2	0	5
Total	26	45	59	100	16.8	24.5

z Chromosome as determined by the pseudomolecules generated by the Potato Genome Sequencing Consortium.

y SNP c2_37964 mapped to two pseudomolecule locations which were both disconcordant with the linkage map positions in DRH and D84 and is therefore included twice in this table.

x Based on the number of segregating markers per chromosome.

**Table 7:** Mapped markers that were not anchored to the pseudomolecules.

	DRH		D84	
	Total unanchored	No. of unique	Total unanchored	No. of unique
Chrm.^z^	markers	unanchored markers	markers	unanchored markers
1	17	16	14	13
2	2	2	3	3
3	10	5	37	32
4	13	9	19	15
5	7	5	5	3
6	27	10	34	17
7	6	4	18	16
8	6	2	37	33
9	9	6	25	22
10	13	8	20	15
11	15	12	17	14
12	7	7	7	7
Total	132	86	236	190

z Chromosome as determined by the pseudomolecules generated by the Potato Genome Sequencing Consortium.

**Figure 3:**
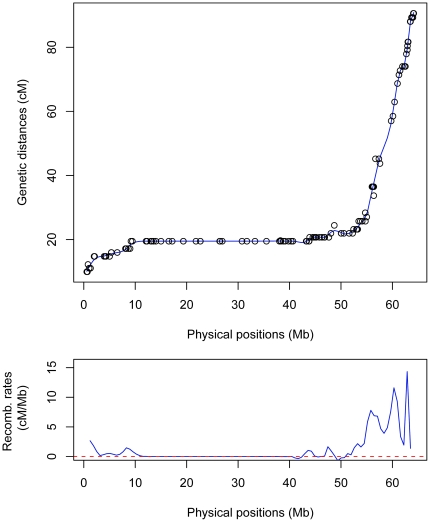
Graph of chromosome 4 (D84) showing the genetic location (cM) and the physical position (Mb) of 204 markers, and the estimated local recombination.

**Figure 4:**
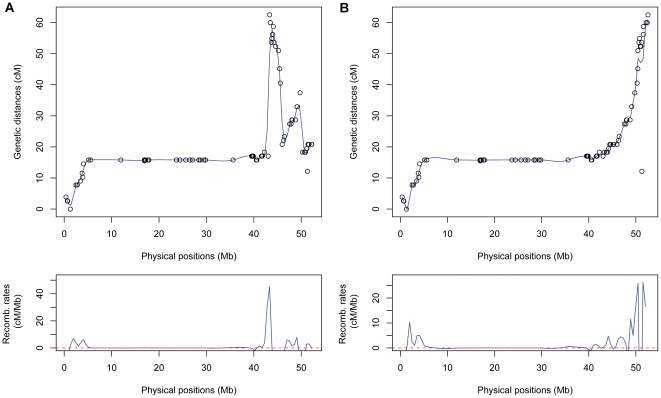
Graph of chromosome 10 (D84) showing the genetic location (cM) and the physical position (Mb) of 98 markers, and the estimated local recombination. **A.** Chromosome 10 from population D84 demonstrating poor concordance between physical and genetic position. **B.** Chromosome 10 from population D84 after correcting the order and orientation of the superscaffolds in the pseudomolecules.

By comparing the genetic location (cM) with the physical position (Mb) of each marker, we evaluated the concordance between the genetic and the physical maps and estimated genome-wide recombination rates. For most of the chromosomes, the resulting graphs had the expected shape, correlating well with chromosome structure (**Figure 3**). Exceptions included DRH chromosomes 3, 5 and 12 and D84 chromosome 10 which indicated local inversions and/or mis-ordering of the superscaffolds in the potato genome sequence. Further examination of the data confirmed several instances where the orientation and/or order of the superscaffolds were in error (**Table S6**). If data from both populations indicated an error in the superscaffolds, the order was corrected. Correcting these issues in our data sets resulted in improved graphs with the previously mentioned exception of chromosome 5 (**Figure 4** **, Figure S3 and Figure S4**). As expected, graphical representation of genome wide recombination rates (RR = cM/Mb) showed greater recombination rates near the ends of the chromosomes (**Figure 3** **, Figure S3 and Figure S4**).

The results presented here demonstrate the utility of the Infinium 8303 Potato Array for mapping studies, providing excellent coverage of the potato genome (633 to 641 Mb) with over 4,400 mapped markers. The high-throughput nature of the array coupled with greater genome coverage than other marker types, make this a valuable tool for Quantitative Trait Locus (QTL) analysis, Genome Wide Association Studies (GWAS) and map-based gene cloning. Furthermore, the close consensus between the two linkage maps and the genome sequence confirmed the high quality of the potato genome sequence. The identification of mis-oriented scaffolds will serve to further refine the genome sequence.

## Materials and Methods

### SNP Array Development

SNPs for the Infinium 8303 Potato Array were selected from the 69,011 high confidence SNPs described in Hamilton et al. [18]. From this set, 3,856 SNPs were selected based on location within a candidate gene of interest as defined by the potato community or a potato genetic marker used in previous mapping studies (**Tables S1 and S2**). The remaining 5,324 SNPs on the array were selected from the 69,011 high confidence SNP set using a custom Perl script to provide coverage of the genome; in total, 650 Mb of the potato genome are represented by SNPs on the array. The Infinium 8303 Potato Array was fabricated by Illumina (Illumina, San Diego, CA).

### Development of a Custom Cluster File for the Infinium 8303 Potato Array

In order to test the utility of the auto-clustering function of the Illumina GenomeStudio software (Illumina, Inc., San Diego, CA), we genotyped DNA from a set of diverse potato clones (443) on an Illumina iScan Reader utilizing the Infinium® HD Assay Ultra (Illumina, Inc., San Diego, CA) and the Infinium 8303 Potato Array. Results were analyzed with the Illumina GenomeStudio software (Illumina, San Diego, CA). Due to variable cluster positioning and quality for each SNP, the software auto-clustering was not used. Clusters for the three marker classes (AA, AB, and BB) were manually positioned within GenomeStudio to generate a custom cluster file, available at http://solcap.msu.edu/potato_infinium.shtml. Note that using the broad set of germplasm when determining the cluster positions and assay quality allowed for more accurate determination of the cluster positions. In addition, the quality of each SNP was manually determined and, of the 8,303 SNPs, 637 had low signal intensity, loose clustering, or other assay failures and were removed from future analyses.

### SNP Genotyping and Linkage Mapping

DNA from all parents and progeny was extracted from young leaf tissue using Qiagen DNeasy Plant Mini Kits (Qiagen, Germantown, MD), quantified using the Quant-iT™ PicoGreen® dsDNA Assay Kit (Invitrogen, San Diego, CA) and adjusted to a concentration of 50 ng·µL^−1^. SNP genotyping with the Infinium 8303 Potato Array was performed as described above and the custom cluster file was used to generate genotype scores for the D84 and DRH mapping populations in GenomeStudio (Illumina, San Diego, CA).

Prior to mapping, SNPs were filtered to remove those which were non-informative including: SNPs that were of low quality (**Table S1**) or that aligned to multiple locations in the superscaffolds generated by the PGSC [15], SNPs for which parental replicated genotypes differed, SNPs for which one or both parents lacked a genotype, SNPs for which DM appeared to be heterozygous, SNPs for which both parents were homozygous (AA×AA or AA×BB), SNPs with a no-call rate of ≥12% (greater than 10 progeny with missing genotypes), SNPs for which the progeny showed no or highly distorted segregation (based on chi-square tests with Bonferroni correction for multiple comparisons) and SNPs which co-segregated with other SNPs based on pairwise comparisons. The remaining SNPs were mapped using JoinMap4 [26]. The data were entered as a cross-pollinated population type with <lmxll> segregation and markers were grouped by regression mapping using Haldane's function. Markers assigned to linkage groups had a minimum LOD score of 3 and a maximum of 10.

A custom version of the version 2.1.10 pseudomolecules was made to remove the large, Mb-sized gaps on a subset of the chromosomes (1, 2, 5 and 12) that reflected estimates of the centromeric gaps. These gaps were resized to the standard gap size of 50 kb. The 8303 SNPs were then aligned to the customized version 2.1.10 pseudomolecules with the est2genome model within exonerate version 2.2.0 [27] using 50 bp of context sequence on both sides of the SNP, a minimum intron size of 10 and a maximum intron size of 15,000. Alignments were required to have greater than 95% sequence identity, greater than 95% coverage, no insertions or deletions, and two or fewer alignments meeting these criteria per SNP. Linkage groups (LGs) generated in JoinMap4 were then aligned to the custom version of the pseudomolecules. If there was a discrepancy between the marker position on the linkage map and the corresponding SNP position on the pseudomolecules, the marker was examined in GenomeStudio to see if the graph of the genotypes indicated the possibility of a paralog.

Genome-wide recombination rates were estimated from the comparison of the linkage maps and the potato pseudomolecules. At any given nucleotide coordinate (SNP marker), the recombination rate was calculated by locally adjusting a polynomial curve to the plot of genetic versus physical distances. This was performed using the MareyMap packages, an R-based tool that uses Tcl/Tk to build the graphical interface [28]. The plots were generated with the graphical interface MareyMapGUI and the slope of the curve was obtained using the “loess” (or lowess for LOcally WEighted Scatterplot Smoothing) interpolation method.

## Supporting Information

Figure S1 **SNP and gene frequency distribution across potato chromosomes.** Frequency is expressed in number of occurrences/100 kbp. A total number of 6,955 SNPs from the Infinium 8303 Potato Array (left) and a total number of 6,351 genes (right) were plotted against physical position to assess SNP coverage on chromosomes 1 through 12. The remaining SNPs (1,348) were not graphed because they were either not mapped to the pseudomolecules or mapped to more than one position on the pseudomolecules. If a gene did not have an associated SNP (1,952) it was not graphed (see Table S1). **A.** Chromosomes 1 through 6. **B.** Chromosomes 7 through 12.
(TIFF)

Figure S2 **Pedigree of potato breeding line 84SD22.**
(TIFF)

Figure S3 **Graphs of the 12 DRH chromosomes showing the genetic location (cM) and the physical position (Mb) of markers, and the estimated local recombination.** Physical marker position (based on corrected superscaffold ordering and orientation) was plotted against genetic marker position to identify areas of discordance between the two (as indicated by peaks and valleys in the graphs). Global recombination rates (cM/Mb) were plotted against physical position to identify areas of higher and lower recombination.
(TIFF)

Figure S4 **Graphs of the 12 D84 chromosomes showing the genetic location (cM) and the physical position (Mb) of markers, and the estimated local recombination.** Physical marker position (based on corrected superscaffold ordering and orientation) was plotted against genetic marker position to identify areas of discordance between the two (as indicated by peaks and valleys in the graphs). Global recombination rates (cM/Mb) were plotted against physical position to identify areas of higher and lower recombination.
(TIFF)

Table S1 **Metadata for the Infinium 8303 Potato Array.** SNP ID numbers with their corresponding superscaffold ID, superscaffold position, pseudomolecule ID, pseudomolecule position, gene and annotation (where applicable), SNP quality call and SNP context sequence. SNPs were manually curated to determine the quality. A SNP was discarded based on low signal intensity, loose clustering, or other assay failures. The context sequence represents the sequences submitted to Illumina. For some SNPs the reverse complement was used in the assay. UM = unmapped; a SNP could not be mapped to the superscaffolds or the pseudomolecules, MM = multiple mapping, a SNP mapped to more than one location on the superscaffolds and pseudomolecules; NG = the SNP is not in a gene.
(XLSX)

Table S2 **List of potato genes containing SNPs on the Infinium 8303 Potato Array.** PGSC gene ID numbers for genes that are represented on the Infinium 8303 Potato Array, the number of SNPs within each gene and the PGSC gene annotation.
(XLSX)

Table S3 **Number of SNPs removed prior to mapping.** A list of the categories of SNPs removed prior to mapping and the number of SNPs removed for each category.
(XLSX)

Table S4 **Comparison of linkage group sizes between populations DRH and D84.** Size differences between linkage groups in populations DRH and D84 demonstrating reduced recombination in D84.
(XLSX)

Table S5 **List of disconcordant markers.** A list of markers for which chromosome assignment based on the genetic maps differs from the chromosome assignment based on the PGSC pseudomolecules. Also included are marker ID numbers, segregation status, annotation and the possibility of a paralog for the marker (based on GenomeStudio graphs).
(XLSX)

Table S6 **List of superscaffolds with mis-alignments.** Superscaffolds that appear to have mis-alignments based on comparison with the genetic maps.
(XLSX)
