# Supplementary figures and images for: Integration of Two Diploid Potato Linkage Maps with the Potato Genome Sequence
Source: PLoS One. 2012 Apr 27;7(4):e36347. doi: 10.1371/journal.pone.0036347 (PMC3338666; doi:10.1371/journal.pone.0036347)

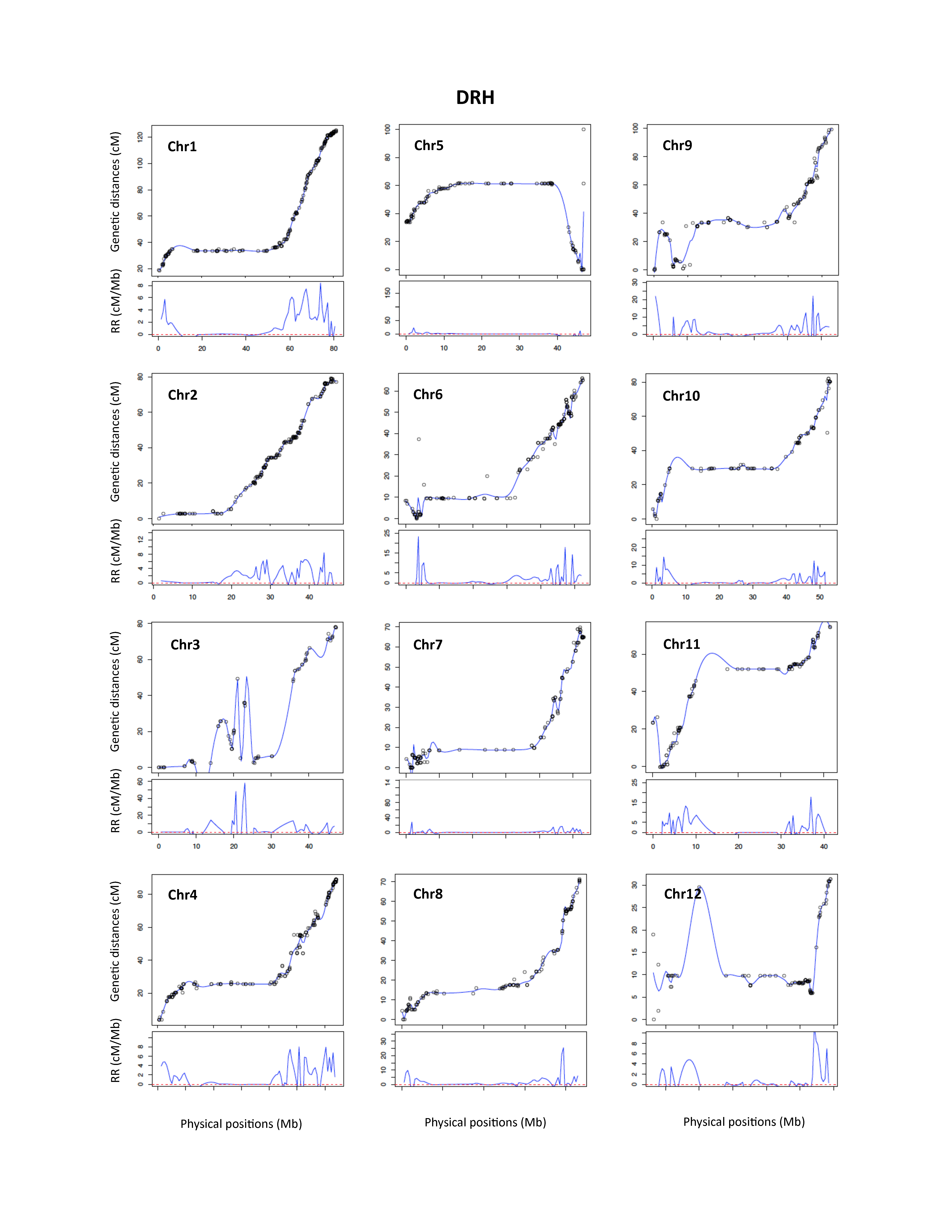

Supplement: Figure S3 — Graphs of the 12 DRH chromosomes showing the genetic location (cM) and the physical position (Mb) of markers, and the estimated local recombination. Physical marker position (based on corrected superscaffold ordering and orientation) was plotted against genetic marker position to identify areas of discordance between the two (as indicated by peaks and valleys in the graphs). Global recombination rates (cM/Mb) were plotted against physical position to identify areas of higher and lower recombination. (TIFF) [file pone.0036347.s003.tiff]

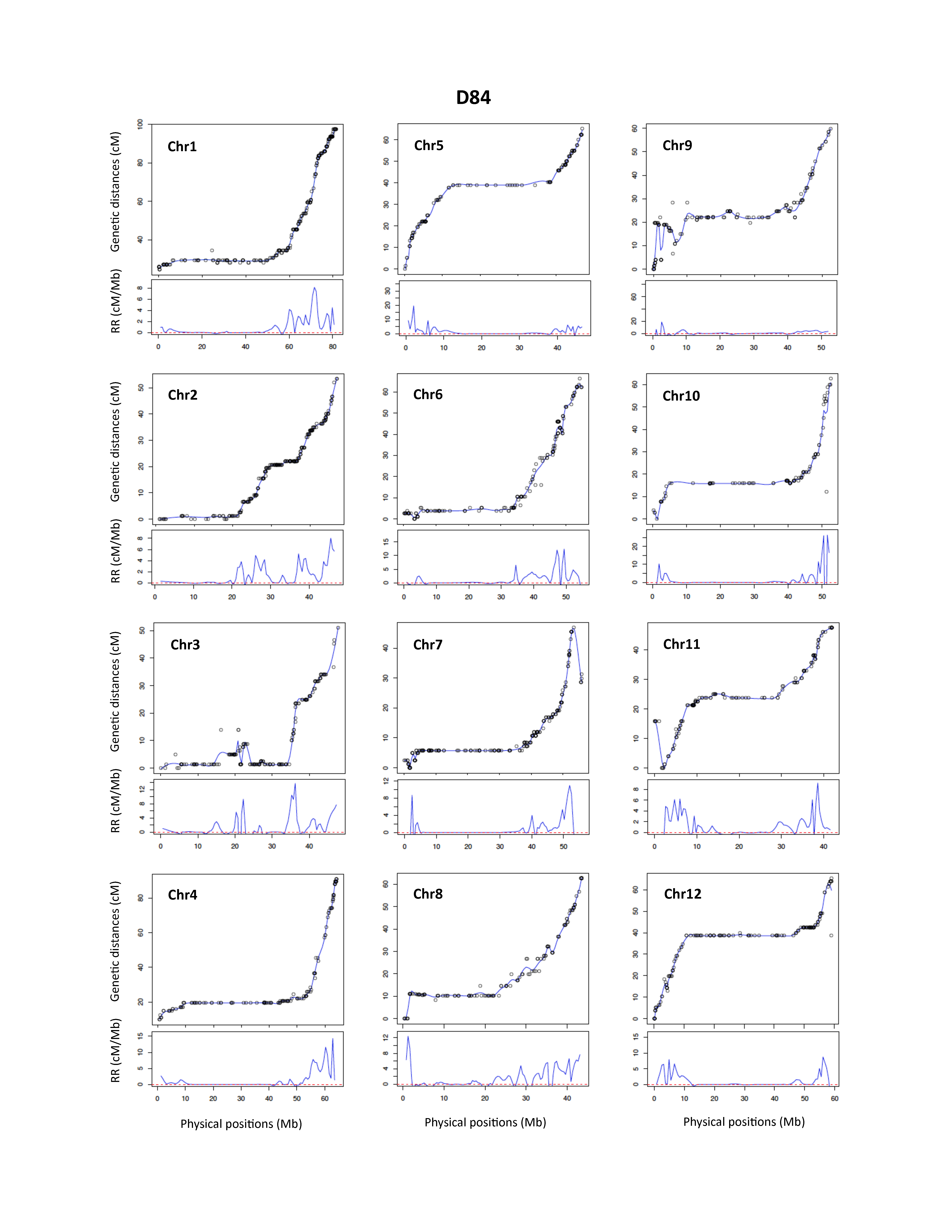

Supplement: Figure S4 — Graphs of the 12 D84 chromosomes showing the genetic location (cM) and the physical position (Mb) of markers, and the estimated local recombination. Physical marker position (based on corrected superscaffold ordering and orientation) was plotted against genetic marker position to identify areas of discordance between the two (as indicated by peaks and valleys in the graphs). Global recombination rates (cM/Mb) were plotted against physical position to identify areas of higher and lower recombination. (TIFF) [file pone.0036347.s004.tiff]
